# InSe/Te van der Waals Heterostructure as a High-Efficiency Solar Cell from Computational Screening

**DOI:** 10.3390/ma14143768

**Published:** 2021-07-06

**Authors:** Zechen Ma, Ruifeng Li, Rui Xiong, Yinggan Zhang, Chao Xu, Cuilian Wen, Baisheng Sa

**Affiliations:** 1 Multiscale Computational Materials Facility, and Key Laboratory of Eco-Materials Advanced Technology, College of Materials Science and Engineering, Fuzhou University, Fuzhou 350100, China; mazc666@163.com (Z.M.); liruifeng1603@126.com (R.L.); 201810004@fzu.edu.cn (R.X.); 2 College of Materials, Xiamen University, Xiamen 361005, China; ygzhang@xmu.edu.cn; 3 Xiamen Talentmats New Materials Science & Technology Co., Ltd., Xiamen 361015, China; xuchao@talentmats.com

**Keywords:** van der Waals heterostructure, InSe, tellurene, first-principle calculations, solar cell

## Abstract

Designing the electronic structures of the van der Waals (vdW) heterostructures to obtain high-efficiency solar cells showed a fascinating prospect. In this work, we screened the potential of vdW heterostructures for solar cell application by combining the group III–VI MX_A_ (M = Al, Ga, In and X_A_ = S, Se, Te) and elementary group VI X_B_ (X_B_ = Se, Te) monolayers based on first-principle calculations. The results highlight that InSe/Te vdW heterostructure presents type-II electronic band structure feature with a band gap of 0.88 eV, where tellurene and InSe monolayer are as absorber and window layer, respectively. Interestingly, tellurene has a 1.14 eV direct band gap to produce the photoexcited electron easily. Furthermore, InSe/Te vdW heterostructure shows remarkably light absorption capacities and distinguished maximum power conversion efficiency (PCE) up to 13.39%. Our present study will inspire researchers to design vdW heterostructures for solar cell application in a purposeful way.

## 1. Introduction

Van der Waals (vdW) heterostructures are stacked by two or more two-dimensional (2D) materials with only vdW interaction in their interlayers but no surface dangling bonds [1], which were widely used in vertical field-effect transistors [2], wearable and biocompatible electronics [3], photodetectors [4], photovoltaics [5,6,7], light-emitting devices (LEDs) [8], and so on. Because vdW force in the interlayer is a long-range weak interaction, the heterostructures can be formed under the existence of large lattice mismatch among the monolayers [9]. Furthermore, vdW heterostructures can combine the excellent properties of the monolayers [10]. Under the interlayer coupling in vdW heterostructures, they can also exhibit novel characteristics that their components do not possess [7,11,12,13]. Designing type-II vdW heterostructures for solar cells through band-structure engineering by using calculations is an efficient way, such as graphene/GaAs [5], Ti_2_CO_2_/Zr_2_CO_2_ [14] and GaSe/GaTe heterostructures [15]. Generally, a vdW heterostructure based high-efficiency solar cell consists of two parts: a absorption layer with a small band gap (~1.2–1.6 eV [16]) and strong light absorption capacity, and a window layer with a large band gap and high transparency for the incident light [17,18]. Besides, the high carrier mobility and the direct band gap of the absorption layer that generates photo-generated electrons are also beneficial for improving the efficiency of solar cells [14,15]. Due to thickness and atomically sharp interfaces, light-generated carriers can be effectively separated in vdW heterostructures. Therefore, the probability of electron-hole recombination is very low, and the efficiency is high [17]. For instance, a 2D WSe_2_-MoS_2_ lateral p-n heterojunction with a power conversion efficiency (PCE) of 2.56% under AM1.5G illumination was designed, which can help develop the next-generation photovoltaics [19]. Hence, finding vdW heterostructures with suitable band gaps and light absorption abilities to obtain high solar energy efficiency is of great interest and importance.

On the other hand, the group III–VI compounds represented by InSe are a kind of layered hexagonal lattice semiconductor [20,21,22]. The layers of them are connected by vdW force without dangling bonds. Excitingly, 2D InSe was successfully prepared experimentally, which exhibits high electron mobility, quantum Hall effect, and anomalous optical response [23]. Moreover, the 2D InSe related vdW heterostructures combined with another layers such as graphene [24], black phosphorus [25], C_3_N_4_ [26], SiGe [7], or III–VI monolayers [15,27] attracted remarkable attention for high-performance electronic and optoelectronic devices. Recently, the 1T-MoS_2_-like phase *T*-Se and *α*-Te were successfully obtained in the laboratory [28,29]. The III–VI monolayers and *T*-Se, *α*-Te are all P63/mmc lattice semiconductors with great optical properties and high carrier mobility [15,30]. Theoretically, the selenene and tellurene are evaluated as indirect band gaps of 1.16 and 1.11 eV [30], respectively, which may be converted to direct band gaps after the formation of vdW heterostructures [31]. Therefore, it is highly desirable to build group III–VI/selenene and III–VI/tellurene vdW heterostructures, which are good candidates for the absorption layers for solar cell application.

In this work, we established the MX_A_/X_B_ vdW heterostructures by combining MX_A_ (M = Al, Ga, In and X_A_ = S, Se, Te) and X_B_ (X_B_ = Se, Te) monolayers. Based on first-principles calculations, we unraveled the electronic structure of each monolayer and heterostructure. Then, according to the energy band requirement of solar cells, InSe/Te vdW heterostructure was screened out for the further study. Our results demonstrated that InSe/Te vdW heterostructure shows type-II electronic band structure feature whose tellurene as absorber layer exhibits 1.14 eV direct HSE band gap, exhibiting distinguished light absorption capacities. Moreover, the corresponding maximum PCE can reach up to 13.39%, which indicates that InSe/Te vdW heterostructure has great potential for high-efficiency solar cells.

## 2. Materials and Methods

The first-principles calculations were based on density functional theory (DFT) using the Vienna ab initio simulation package (VASP) [32,33,34,35] in conjunction with the projector augmented wave (PAW) pseudopotentials [32,36]. The calculation models and results were dealt with the ALKEMIE platform [37]. The generalized gradient approximation (GGA) [38] of Perdew–Burke–Ernzerhof (PBE) [39] pseudopotentials were selected to descried the exchange correlation interactions between electrons. Our work conducted by using van der Waals (vdW) interaction to form a heterostructure with two monolayers. Since the weak interaction is difficult to be evaluated by traditional exchange and correlation potentials [40], the optB86b-vdW functional [41] was used to include the weak interaction in all the calculations. For the lattice optimization, the cutoff energy and the convergence criteria for energy were set to 500 eV and 10^−5^ eV·atom^−1^, respectively. We maintained a sufficiently large vacuum space (~20 Å vacuum for each layer) in the z-direction, and a proper distance (~3 Å) between the two layers in order to ensure that there was only vdW interaction between the different layers and no significant interaction among the repeating units in the vertical direction. In geometric optimizations and static self-consistent calculations, *k*-sampling was performed using 9 × 9 × 1 points by the Monkhorst–Pack [42] mesh. The Heyd–Scuseria–Ernzerhof (HSE06) [41] hybrid functional was used to evaluate the electronic band gaps.

## 3. Results and Discussion

### 3.1. Geometry and Stability

**Figure 1:**
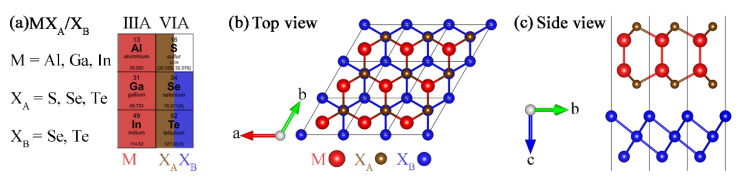
(**a**) Position in periodic table of elements for MX_A_/X_B_ (M = Al, Ga, In, X_A_ = S, Se, Te and X_B_ = Se, Te) heterostructures. (**b**) top- and (**c**) side-views of optimized structure of MX_A_/X_B_ heterostructures.

The MX_A_ (M = Al, Ga, In and X_A_ = S, Se, Te) and X_B_ (X_B_ = Se, Te) monolayers, where the positions of the elements in the periodic table are shown in Figure 1a, are crystallized in the space group of *P63/mmc* with a honeycomb hexagonal structure [30,43]. Table S1 lists the optimized lattice constant, bond length and band gaps for the monolayers, where the results are in good agreement with the previous reports [44,45]. The lattice constants of most of the MX_A_ and X_B_ monolayers are close to each other; for instance, the lattice differences between AlSe monolayer and selenene, InSe monolayer and tellurene, are 0.062 and 0.144 Å, respectively. The corresponding mismatches are 1.6% and 3.4%, respectively. The well matched crystalline nature is beneficial for the assembly of van der Waals (vdW) heterostructures, as illustrated in Figure 1b,c.

**Figure 2:**
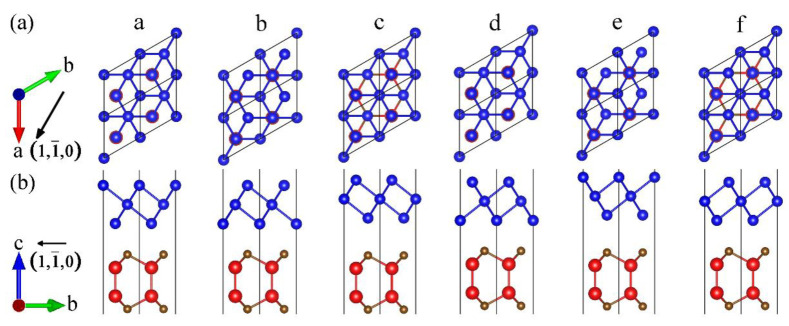
Top (**a**) and side (**b**) views of MX_A_/X_B_ heterostructures with various configurations. Red, brown, blue balls indicate the M, X_A_, X_B_ atoms, respectively.

We established the MX_A_/X_B_ vdW heterostructures by placing the X_B_ monolayers on the top of MX_A_ monolayers. There are six possible stacking configurations of the heterostructures [46], named configurations (a) to (f) in Figure 2. In configuration (a), X_A_ atom of the MX_A_ monolayer is placed below the bottom X_B_ atom. While in configurations (b) or (c), X_A_ atom is located in the bottom of the middle or upper X_B_ atom. At the same time, we can also regard configurations (b) and (c) as the shifting of the X_B_ monolayer in configuration (a) along the [1$\bar{1}$0] direction of 1/3 and 2/3 *a*, respectively. Besides, the configurations (d), (e), and (f) can be obtained by flip the X_B_ monolayer of (a), (b), and (c) types around the horizontal plane with an angle of 180°. After structural optimizations for a total of 288 structures of all the MX_A_/X_B_ heterostructures, the energy differences between different configurations, the interlayer distances, lattice constants, and bond lengths are listed in Tables S2–S4. The energy difference Δ*E*_i_ refers to the difference between the corresponding configuration and the most stable configuration, which can be defined as follows [47]:

(1) $$\Delta E_{i}=E_{i}-E_{0}$$

where *E*_i_ is the total energy of each configuration, and *E*_0_ is the total energy of the most stable configuration. The most stable configuration, which has zero Δ*E*_i_, is presented in configurations (b) and (d). Moreover, the calculated total energy of various configurations relies on the interlayer distances and lattice constants [48]. Therefore, configurations (b) and (d) show a lower interlayer distance. Moreover, Figure 2 shows that the atom in the bottom of X_B_ monolayer is not aligned with any atom in MX_A_ monolayer.

To evaluate the thermodynamic stability and interlayer interaction, we calculated the formation energy *E*_f_ and binding energy *E*_b_ for the heterostructures according to the following equations:

(2) $$E_{f}=E_{\mathrm{total}}-E_{{\mathrm{MX}}_{A}}-E_{X_{B}}$$

(3) $$E_{b}=-\frac{E_{\mathrm{total}}-E_{{\mathrm{MX}}_{A}{+X}_{B}}}{A}$$

where *E*_total_ is the total energy of the MX_A_/X_B_ heterostructures. $E_{{\mathrm{MX}}_{A}}$ and $E_{X_{B}}$ represent the total energy of pristine MX_A_ and X_B_ monolayers, respectively. In addition, $E_{{\mathrm{MX}}_{A}{+X}_{B}}$ is the sum of the total energy of the mutually independent MX_A_ and X_B_ monolayers fixed in the corresponding heterostructure lattices, and *A* is the interface area. Table 1 lists the formation and binding energies and other related parameters of the most stable configuration of the MX_A_/X_B_ heterostructures. In addition, most of heterostructures have the negative value of formation energy, which indicates that the reaction of combining monolayers to form these heterostructures is energetically favorable [49]. For example, those of AlTe/Te, GaTe/Te and InSe/Te heterostructures are −280.3, −300.8 and −278.7 meV, respectively. On the other hand, all the heterostructures have the binding energy around ~20 meV/Å^2^, which is the sign of vdW interaction between two monolayers [50].

### 3.2. Electronic Properties

A high-efficiency heterostructure solar cell requires the type-II band structure feature, and the absorption layer has a lower band edge than the window layer, preferably with a direct band gap of 1.2–1.4 eV [16,17]. Figure S1 illustrates the projected band structures and band edge alignments of MX_A_ and X_B_ monolayers by using HSE06 hybrid functional, while Table S1 lists their corresponding PBE and HSE band gaps. For instance, the conduction band minima (CBM) and band gap for InSe monolayer are −4.46 and 2.32 eV, and CBM and band gap for tellurene are −4.49 and 1.09 eV, respectively. In addition, the CBM of tellurene located in Г point is only 0.07 eV higher than the energy of the point where VBM located in valance band.

**Figure 3:**
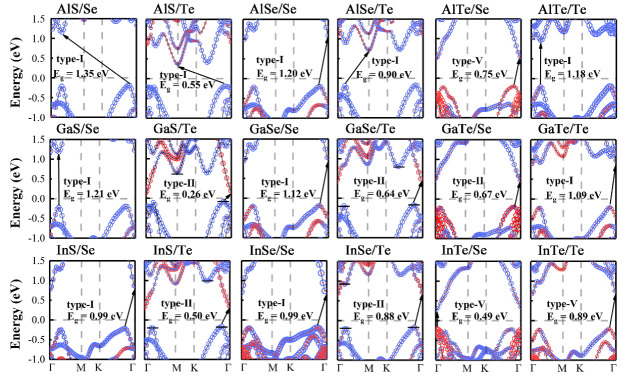
Projected band structures of MX_A_/X_B_ heterostructures by HSE hybrid functional method. Red and blue circles represent projected weight of MX_A_ and X_B_ monolayers, respectively.

**Figure 4:**
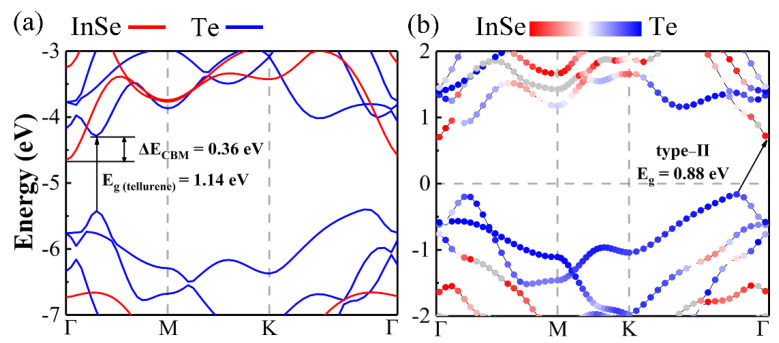
(**a**) Overlap band structures of mutually independent monolayers fixed in InSe/Te heterostructure and (**b**) projected band structure of InSe/Te heterostructure via HSE06 hybrid functional method.

Figure 3 illustrates the HSE band structures of all the MX_A_/X_B_ heterostructures. GaS/Te, GaSe/Te, InS/Te and InSe/Te are all type-II heterostructures with tellurene as the absorption layer. The black short lines mark the corresponding positions of the CBM and VBM of tellurene. In the band structures of GaSe/Te, InS/Te, and InSe/Te, there are two lines in valence bands because their energy levels are similar. The overlap band structures of mutually independent monolayers fixed in InSe/Te heterostructure and the projected HSE band structure of InSe/Te vdW heterostructure are illustrated in Figure 4. Tellurene exhibits the direct band gap of 1.14 eV. And there is 0.36 eV conduction band offset (CBO) between InSe monolayer and tellurene to separate charges [51]. Therefore, InSe/Te vdW heterostructure has the suitable band structure for solar cells.

### 3.3. Solar Cell Applications

To further evaluate the light absorption capacity and reflectivity of InSe/Te vdW heterostructure, we calculated the absorption coefficient and reflectivity by the following formula [52]:

(4) $$n(\lambda )=\frac{1}{\sqrt{2}}\sqrt{\varepsilon_{1}\left(\lambda \right)+\sqrt{\varepsilon_{1}{}^{2}\left(\lambda \right)+\varepsilon_{2}{}^{2}\left(\lambda \right)}}$$

(5) $$\kappa \left(\lambda \right)=\frac{1}{\sqrt{2}}\sqrt{-\varepsilon_{1}\left(\lambda \right)+\sqrt{\varepsilon_{1}{}^{2}\left(\lambda \right)+\varepsilon_{2}{}^{2}\left(\lambda \right)}}$$

(6) $$\alpha \left(\lambda \right)=\frac{2\pi \varepsilon_{2}}{\lambda }$$

(7) $$R\left(\lambda \right)=\frac{{\left(n-1\right)}^{2}+\kappa^{2}}{{\left(n+1\right)}^{2}+\kappa^{2}}$$

where *λ* is the photon wavelength, *ԑ*_1_ and *ԑ*_2_ are the real and imaginary parts of the dielectric function, respectively, and *n*(*λ*), *к*(*λ*) are the refractive index and the extinction coefficient, respectively. *α*(*λ*) and *R*(*λ*) are the absorption coefficient and reflectivity, respectively.

**Figure 5:**
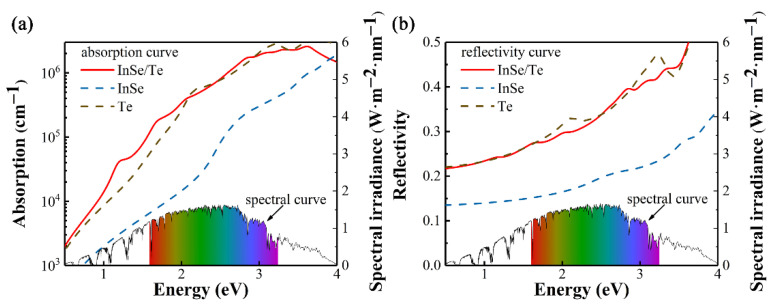
(**a**) Calculated optical absorption coefficients as well as (**b**) the reflectivity of InSe/Te heterostructure, InSe monolayer, and tellurene. Curve in bottom indicates reference solar spectral irradiance, and colorful background represents visible light area [55].

The absorption coefficients and reflectivity curves of InSe/Te heterostructure, InSe monolayer and tellurene are shown in Figure 5. Herein, tellurene as the absorption layer exhibits the high absorption coefficient about 10^5^ to 10^6^ cm^−1^ in the visible light, which can be comparable with that of bulk WS_2_ and WSe_2_ used in efficient single junction solar cell [53]. The InSe monolayer as the window layer is required high transparency for the incident light, which means low absorption coefficient and reflectivity [17,54]. The InSe monolayer has an absorption coefficient about one order of magnitude lower than that of tellurene, and the reflectivity of it is about 0.13 to 0.34 in the range of 0 to 4 eV photon energy. This result can be compared with that of the Janus WSeTe monolayer used as buffer layer [54].

**Figure 6:**
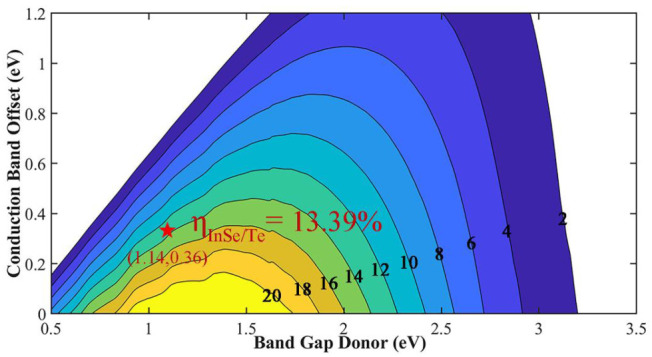
Simulated solar cell power conversion efficiency (PCE) *η* for InSe/Te heterostructure (marked as the red star).

To more intuitively evaluate the solar energy conversion ability of InSe/Te vdW heterostructure, we evaluated the power conversion efficiency (PCE) *η* in the limit of 100% external quantum efficiency (EQE) by the following equation [6,51]:

(8) $$\eta =\frac{0.65(E_{g}-\Delta E_{c}-0.3)\int_{E_{g}}^{\infty }\frac{P(\hbar \omega )}{\hbar \omega }d(\hbar \omega )}{\int_{0}^{\infty }P(\hbar \omega )d(\hbar \omega )}$$

where 0.65 is the band-fill factor, *P*(*ћω*) is the AM1.5 solar energy flux at the value of *ћω* for photon energy, *E*_g_ is the band gap of the donor, Δ*E*_c_ is the conduction band offset between the donor and acceptor, and the (*E*_g_ − Δ*E*_c_ − 0.3) term is an estimation of the maximum open circuit voltage. For this formula, the smaller Δ*E*_c_ means the greater value of PCE. Additionally, it requires a suitable *E*_g_, because if the band gap of the donor is higher, the open circuit voltage will be better. However, the higher band gap will reduce the amount of photons that can be absorbed, which will reflect in the decrease of short circuit current. Here, the maximum PCE of InSe/Te vdW heterostructure is calculated to 13.39%, which is highlighted as red star in Figure 6. To show the uniqueness of InSe/Te vdW heterostructure, the PCE calculated by the same method for other 2D heterostructure solar cells are listed in Table 2. Therefore, we infer that the InSe/Te vdW heterostructure is a potential candidate for the high-efficiency solar cell application.

## 4. Conclusions

In summary, we established the vdW heterostructures by combining MX_A_ (M = Al, Ga, In and X_A_ = S, Se, Te) and X_B_ (X_B_ = Se, Te) monolayers. Based on first-principles calculations, the stability and interlayer force of these heterostructures were demonstrated by the formation and binding energy. From screening, the InSe/Te vdW heterostructure shows type-II electronic band structure feature with a band gap of 0.88 eV, where the tellurene as absorber layer with a direct band gap about 1.14 eV could produce the photoexcited electron easily. In addition, tellurene and InSe monolayer respectively exhibit high absorption coefficient and low reflectivity. Furthermore, the maximum power conversion efficiency (PCE) of InSe/Te vdW heterostructure can reach up to 13.39%. Very recently, multilayer InSe/Te vdW heterostructure was experimentally observed and showed potential application in electronic and optoelectronic devices [57]. We believed that monolayer InSe/Te vdW heterostructure can be experimentally realized and show better performance. Our present research not only finds a novel type-II heterostructure for high-efficiency solar cell, but also further guides the design of more 2D vdW semiconductors for photovoltaic materials.

## Figures and Tables

**Table 1:** Most stable configurations, lattice constants *a* (Å), formation energies *E*_f_ (meV), binding energies *E*_b_ (meV/Å^2^), PBE, and HSE band gaps $E_{g}^{\mathrm{PBE}}$ (eV) and $E_{g}^{\mathrm{HSE}}\;$ (eV), and band edge alignment types for MX_A_/X_B_ vdW heterostructures.

System	Configuration	a	*E*_f_	*E*_b_	$E_{g}^{\mathrm{PBE}}$	$E_{g}^{\mathrm{HSE}}$	Type
AlS-Se	d	3.627	−174.0	20.7	1.00	1.35	Ι
AlS-Te	d	3.822	492.3	20.4	0.15	0.55	Ι
AlSe-Se	d	3.744	−246.9	20.9	0.76	1.20	Ι
AlSe-Te	d	3.935	58.5	20.4	0.52	0.90	Ι
AlTe-Se	b	3.965	−67.9	24.1	0.43	0.75	V
AlTe-Te	d	4.129	−280.3	20.5	0.83	1.18	Ι
GaS-Se	d	3.660	−213.7	21.2	0.76	1.21	Ι
GaS-Te	d	3.863	328.0	22.4	0.00	0.26	II
GaSe-Se	d	3.764	−250.0	21.5	0.60	1.12	Ι
GaSe-Te	d	3.963	−24.3	21.8	0.20	0.64	II
GaTe-Se	b	3.980	−74.8	25.6	0.36	0.67	II
GaTe-Te	d	4.141	−300.8	21.4	0.58	1.09	Ι
InS-Se	b	3.829	−206.7	21.4	0.60	0.99	Ι
InS-Te	d	4.024	−164.8	21.7	0.16	0.50	II
InSe-Se	b	3.921	−107.9	22.7	0.44	0.99	Ι
InSe-Te	d	4.112	−278.7	21.5	0.39	0.88	II
InTe-Se	b	4.124	178.0	29.0	0.20	0.49	V
InTe-Te	b	4.290	−309.5	21.7	0.44	0.89	V

**Table 2:** Calculated maximum power conversion efficiency (PCE) (%) of some recently reported 2D heterostructure solar cells.

System	PCE	References
InSe/Te	13.39	This work
GaTe/InS, GaTe/GaSe	11.52, 18.39	[15]
Ti_2_CO_2_/Zr_2_CO_2_	22.74	[14]
phosphorene/MoS_2_	16–18	[56]
PCBM/CBN	10–20	[6]

## Data Availability

The data presented in this study are available on request from the corresponding authors.

## Supplementary Materials

The following are available online at https://www.mdpi.com/article/10.3390/ma14143768/s1, Figure S1: Projected band structures of (a) AlS, (b) AlSe, (c) AlTe, (d) GaS, (e) GaSe, (f) GaTe, (g) InS, (h) InSe, (i) InTe, (j) Se and (k) Te monolayers by HSE06 hybrid functional method. The red, brown, blue circles represent the projected specific gravity of M, X_A_, X_B_ atoms, respectively. The first Brillouin zone with high-symmetry points are shown in the inset of (a). (l) The band edge alignments of these monolayers, Table S1: the lattice constants *a* (Å), the M-M, M-X_A_ and X_B_-X_B_ (M = Al, Ga, In, X_A_ = S, Se, Te and X_B_ = Se, Te) bond lengths *L*_M-M_ (Å), *L*_M-XA_ (Å) and *L*_XB-XB_ (Å), the PBE and HSE band gaps $E_{g}^{\mathrm{PBE}}$ (eV), $E_{g}^{\mathrm{HSE}}$ (eV) for the MX_A_ and X_B_ monolayers, Table S2: the energy differences Δ*E* (meV) and interlayer distances *d* (Å) as well as the lattice constants *a* (Å) and bond lengths *L* (Å) of various configurations for AlX_A_/X_B_ vdW heterostructures, Table S3: the energy differences Δ*E* (meV) and interlayer distances *d* (Å) as well as the lattice constants *a* (Å) and bond lengths *L* (Å) of various configurations for GaX_A_/X_B_ vdW heterostructures, Table S4: the energy differences Δ*E* (meV) and interlayer distances *d* (Å) as well as the lattice constants *a* (Å) and bond lengths *L* (Å) of various configurations for InX_A_/X_B_ vdW heterostructures.

## References

[1] Geim A.K., Grigorieva I.V. (2013). Van der Waals heterostructures. Nature.

[2] Liu Y., Weiss N.O., Duan X., Cheng H.-C., Huang Y., Duan X. (2016). Van der Waals heterostructures and devices. Nat. Rev. Mater..

[3] Cheng W., Zhou Z., Pan M., Yang H., Xie Y., Wang B., Zhan Q., Li R.-W. (2019). Stretchable spin valve with strain-engineered wrinkles grown on elastomeric polydimethylsiloxane. J. Phys. D Appl. Phys..

[4] Wang X., Xia F. (2015). Van der Waals heterostructures: Stacked 2D materials shed light. Nat. Mater..

[5] Li X., Chen W., Zhang S., Wu Z., Wang P., Xu Z., Chen H., Yin W., Zhong H., Lin S. (2015). 18.5% efficient graphene/GaAs van der Waals heterostructure solar cell. Nano Energy.

[6] Bernardi M., Palummo M., Grossman J.C. (2012). Semiconducting Monolayer Materials as a Tunable Platform for Excitonic Solar Cells. ACS Nano.

[7] Eren I., Ozen S., Sozen Y., Yagmurcukardes M., Sahin H. (2019). Vertical van der Waals Heterostructure of Single Layer InSe and SiGe. J. Phys. Chem. C.

[8] Withers F., Pozo-Zamudio O.D., Mishchenko A., Rooney A.P., Gholinia A., Watanabe K., Taniguchi T., Haigh S.J., Geim A.K., Tartakovskii A.I. (2015). Light-emitting diodes by band-structure engineering in van der Waals heterostructures. Nat. Mater..

[9] Koma A. (1992). Van der Waals epitaxy—A new epitaxial growth method for a highly lattice-mismatched system. Thin Solid Films.

[10] Pierucci D., Henck H., Avila J., Balan A., Naylor C.H., Patriarche G., Dappe Y.J., Silly M.G., Sirotti F., Johnson A.T. (2016). Band alignment and minigaps in monolayer MoS_2_-graphene van der Waals heterostructures. Nano Lett..

[11] Jin C., Ma E.Y., Karni O., Regan E.C., Wang F., Heinz T.F. (2018). Ultrafast dynamics in van der Waals heterostructures. Nat. Nanotechnol..

[12] Liu K., Zhang L., Cao T., Jin C., Qiu D., Zhou Q., Zettl A., Yang P., Louie S.G., Wang F. (2014). Evolution of interlayer coupling in twisted molybdenum disulfide bilayers. Nat. Commun..

[13] Duong D.L., Yun S.J., Lee Y.H. (2017). Van der Waals Layered Materials: Opportunities and Challenges. ACS Nano.

[14] Zhang Y., Xiong R., Sa B., Zhou J., Sun Z. (2021). MXenes: Promising donor and acceptor materials for high-efficiency heterostructure solar cells. Sustain. Energy Fuels.

[15] Chen J., He X., Sa B., Zhou J., Xu C., Wen C., Sun Z. (2019). III–VI van der Waals heterostructures for sustainable energy related applications. Nanoscale.

[16] Shockley W., Queisser H.J. (1961). Detailed balance limit of efficiency of p-n junction solar cells. J. Appl. Phys..

[17] Das S., Pandey D., Thomas J., Roy T. (2019). The role of graphene and other 2D materials in solar photovoltaics. Adv. Mater..

[18] Fonash S.J. (1981). Solar Cell Device Physics.

[19] Tsai M.L., Li M.Y., Retamal J.R.D., Lam K.T., Lin Y.C., Suenaga K., Chen L.J., Liang G., Li L.J., He J.H. (2017). Single atomically sharp lateral monolayer p-n heterojunction solar cells with extraordinarily high power conversion efficiency. Adv. Mater..

[20] Tan C., Cao X., Wu X.J., He Q., Yang J., Zhang X., Chen J., Zhao W., Han S., Nam G.H. (2017). Recent advances in ultrathin two-dimensional nanomaterials. Chem. Rev..

[21] Kuhn A., Chevy A., Chevalier R. (1975). Crystal structure and interatomic distances in GaSe. Phys. Status Solidi.

[22] Gouskov A., Camassel J., Gouskov L. (1982). Growth and characterization of III–VI layered crystals like GaSe, GaTe, InSe, GaSe_1−x_Te_x_ and Ga_x_In_1−x_Se. Prog. Cryst. Growth Charact..

[23] Bandurin D.A., Tyurnina A.V., Yu G.L., Mishchenko A., Zolyomi V., Morozov S.V., Kumar R.K., Gorbachev R.V., Kudrynskyi Z.R., Pezzini S. (2017). High electron mobility, quantum Hall effect and anomalous optical response in atomically thin InSe. Nat. Nanotechnol..

[24] Mudd G.W., Svatek S.A., Hague L., Makarovsky O., Kudrynskyi Z.R., Mellor C.J., Beton P.H., Eaves L., Novoselov K.S., Kovalyuk Z.D. (2015). High broad-band photoresponsivity of mechanically formed InSe-graphene van der Waals heterostructures. Adv. Mater..

[25] Zhao S., Wu J., Jin K., Ding H., Li T., Wu C., Pan N., Wang X. (2018). Highly polarized and fast photoresponse of black phosphorus-InSe vertical p-n heterojunctions. Adv. Funct. Mater..

[26] He C., Zhang J.H., Zhang W.X., Li T.T. (2019). Type-II InSe/g-C_3_N_4_ heterostructure as a high-efficiency oxygen evolution reaction catalyst for photoelectrochemical water splitting. J. Phys. Chem. Lett..

[27] Yan F., Zhao L., Patane A., Hu P., Wei X., Luo W., Zhang D., Lv Q., Feng Q., Shen C. (2017). Fast, multicolor photodetection with graphene-contacted p-GaSe/n-InSe van der Waals heterostructures. Nanotechnology.

[28] Apte A., Bianco E., Krishnamoorthy A., Yazdi S., Rao R., Glavin N., Kumazoe H., Varshney V., Roy A., Shimojo F. (2018). Polytypism in ultrathin tellurium. 2D Mater..

[29] Xing C., Xie Z., Liang Z., Liang W., Fan T., Ponraj J.S., Dhanabalan S.C., Fan D., Zhang H. (2017). 2D nonlayered selenium nanosheets: Facile synthesis, photoluminescence, and ultrafast photonics. Adv. Opt. Mater..

[30] Singh J., Jamdagni P., Jakhar M., Kumar A. (2020). Stability, electronic and mechanical properties of chalcogen (Se and Te) monolayers. Phys. Chem. Chem. Phys..

[31] Peng Q., Guo Z., Sa B., Zhou J., Sun Z. (2018). New gallium chalcogenides/arsenene van der Waals heterostructures promising for photocatalytic water splitting. Int. J. Hydrogen Energy.

[32] Kresse G.G., Furthmüller J.J. (1996). Efficient Iterative Schemes for Ab Initio Total-Energy Calculations Using a Plane-Wave Basis Set. Phys. Rev. B Condens. Matter.

[33] Kresse G., Joubert D. (1999). From ultrasoft pseudopotentials to the projector augmented-wave method. Phys. Rev. B.

[34] Hafner J. (2008). Ab-initio simulations of materials using VASP: Density-functional theory and beyond. J. Comput. Chem..

[35] Kresse G., Hafner J. (1994). Ab initio molecular-dynamics simulation of the liquid-metal-amorphous-semiconductor transition in germanium. Phys. Rev. B Condens. Matter.

[36] Blochl P.E. (1994). Projector augmented-wave method. Phys. Rev. B Condens. Matter.

[37] Wang G., Peng L., Li K., Zhu L., Zhou J., Miao N., Sun Z. (2021). ALKEMIE: An intelligent computational platform for accelerating materials discovery and design. Comput. Mater. Sci..

[38] Perdew J.P., Wang Y. (1992). Accurate and simple analytic representation of the electron-gas correlation energy. Phys. Rev. B Condens. Matter.

[39] Perdew J.P., Burke K., Ernzerhof M. (1998). Generalized Gradient Approximation Made Simple. Phys. Rev. Lett..

[40] Yang X., Sa B., Zhan H., Sun Z. (2017). Electric field-modulated data storage in bilayer InSe. J. Mater. Chem. C.

[41] Klimes J., Bowler D.R., Michaelides A. (2010). Chemical accuracy for the van der Waals density functional. J. Phys. Condens. Matter.

[42] Monkhorst H.J., Pack J.D. (1976). Special points for Brillouin-zone integrations. Phys. Rev. B.

[43] Peng Q., Xiong R., Sa B., Zhou J., Wen C., Wu B., Anpo M., Sun Z. (2017). Computational mining of photocatalysts for water splitting hydrogen production: Two-dimensional InSe-family monolayers. Catal. Sci. Technol..

[44] Demirci S., Avazlı N., Durgun E., Cahangirov S. (2017). Structural and electronic properties of monolayer group III monochalcogenides. Phys. Rev. B.

[45] Zhuang H.L., Hennig R.G. (2013). Single-layer group-III monochalcogenide photocatalysts for water splitting. Chem. Mater..

[46] Peng Q., Wang Z., Sa B., Wu B., Sun Z. (2016). Blue phosphorene/MS_2_ (M = Nb, Ta) heterostructures as promising flexible anodes for lithium-Ion batteries. ACS Appl. Mater. Interfaces.

[47] Yang X., Sa B., Lin P., Xu C., Zhu Q., Zhan H., Sun Z. (2020). Tunable contacts in graphene/InSe van der Waals heterostructures. J. Phys. Chem. C.

[48] Chiu M.H., Zhang C., Shiu H.W., Chuu C.P., Chen C.H., Chang C.Y., Chen C.H., Chou M.Y., Shih C.K., Li L.J. (2015). Determination of band alignment in the single-layer MoS_2_/WSe_2_ heterojunction. Nat. Commun..

[49] Liao J., Sa B., Zhou J., Ahuja R., Sun Z. (2014). Design of high-efficiency visible-light photocatalysts for water splitting: MoS_2_/AlN(GaN) heterostructures. J. Phys. Chem. C.

[50] Bjoerkman T., Gulans A., Krasheninnikov A.V., Nieminen R.M. (2012). van der Waals bonding in layered compounds from advanced density-functional first-principles calculations. Phys. Rev. Lett..

[51] Scharber M.C., Mühlbacher D., Koppe M., Denk P., Waldauf C., Heeger A.J., Brabec C.J. (2006). Design rules for donors in bulk-heterojunction solar cells—Towards 10 % energy-conversion efficiency. Adv. Mater..

[52] Peng Q., Wang Z., Sa B., Wu B., Sun Z. (2016). Electronic structures and enhanced optical properties of blue phosphorene/transition metal dichalcogenides van der Waals heterostructures. Sci. Rep..

[53] Chaurasiya R., Gupta G.K., Dixit A. (2019). Ultrathin Janus WSSe buffer layer for W(S/Se)2 absorber based solar cells: A hybrid, DFT and macroscopic, simulation studies. Sol. Energy Mater. Sol. Cells.

[54] Chaurasiya R., Gupta G.K., Dixit A. (2021). Heterostructure AZO/WSeTe/W(S/Se)_2_ as an efficient single junction solar cell with ultrathin janus WSeTe buffer layer. J. Phys. Chem. C.

[55] (2012). A. G173-03, A.I. West Conshohocken, PA. www.astm.org.

[56] Dai J., Zeng X.C. (2014). Bilayer phosphorene: Effect of stacking order on bandgap and its potential applications in thin-film solar cells. J. Phys. Chem. Lett..

[57] Qin F., Gao F., Dai M., Hu Y., Yu M., Wang L., Feng W., Li B., Hu P. (2020). Multilayer InSe-Te van der Waals Heterostructures with an Ultrahigh Rectification Ratio and Ultrasensitive Photoresponse. ACS Appl. Mater. Interfaces.

