# Supplementary material for: InSe/Te van der Waals Heterostructure as a High-Efficiency Solar Cell from Computational Screening
Source: Materials (Basel). 2021 Jul 6;14(14):3768. doi: 10.3390/ma14143768 (PMC8306732; doi:10.3390/ma14143768)
Supplement: Supplementary file 1 [file materials-14-03768-s001.zip › materials-1255805-SI.pdf]

Electronic Supplementary Material for

# InSe/Te van der Waals Heterostructure as a High-Efficiency Solar Cell from Computational Screening

**Zechen Ma [1,†], Ruifeng Li [1,†], Rui Xiong[1], Yinggan Zhang [2], Chao Xu [3], Cuilian Wen [1,*] and Baisheng Sa [1,*]**

1 Multiscale Computational Materials Facility, and Key Laboratory of Eco-materials Advanced Technology, College of Materials Science and Engineering, Fuzhou University, Fuzhou 350100, P. R. China

2 College of Materials, Xiamen University, Xiamen 361005, P. R. China

3 Xiamen Talentmats New Materials Science & Technology Co., Ltd., Xiamen 361015, P. R. China

* Correspondence: clwen@fzu.edu.cn (C. Wen); bssa@fzu.edu.cn (B. Sa)

† These authors equally contributed to this work.

**Table S1.** The lattice constants $a$ (Å), the M-M, M-$X_A$ and $X_B$-$X_B$ (M = Al, Ga, In, $X_A$ = S, Se, Te and $X_B$ = Se, Te) bond lengths $L_{M-M}$ (Å), $L_{M-X_A}$ (Å) and $L_{X_B-X_B}$ (Å), the PBE and HSE band gaps $E_g^{PBE}$ (eV), $E_g^{HSE}$ (eV) for the $MX_A$ and $X_B$ monolayers

| System | $a$ | $L_{M-M}$ | $L_{M-X_A}$ | $L_{X_B-X_B}$ | $E_g^{PBE}$ | $E_g^{HSE}$ |
|---|---|---|---|---|---|---|
| AlS | 3.563 | 2.588 | 2.320 | | 2.19 | 3.16 |
| AlSe | 3.776 | 2.586 | 2.472 | | 1.97 | 2.97 |
| AlTe | 4.116 | 2.573 | 2.700 | | 1.88 | 2.71 |
| GaS | 3.612 | 2.466 | 2.362 | | 2.41 | 3.46 |
| GaSe | 3.787 | 2.462 | 2.492 | | 1.85 | 2.82 |
| GaTe | 4.096 | 2.459 | 2.696 | | 1.48 | 2.22 |
| InS | 3.893 | 2.800 | 2.556 | | 1.76 | 2.66 |
| InSe | 4.041 | 2.797 | 2.677 | | 1.51 | 2.32 |
| InTe | 4.327 | 2.790 | 2.875 | | 1.43 | 2.16 |
| Se | 3.714 | | | 2.668 | 0.61 | 1.14 |
| Te | 4.185 | | | 3.036 | 0.59 | 1.09 |

**Table S2.** The energy differences Δ*E* (meV) and interlayer distances *d* (Å) as well as the lattice constants *a* (Å) and bond lengths *L* (Å) of various configurations for $AlX_A/X_B$ vdW heterostructures

| System | Configuration | Δ*E* | *d* | *a* | $L_{M\text{-}M}$ | $L_{M\text{-}X_A}$ | $L_{X_A\text{-}X_B}$ |
|---|---|---|---|---|---|---|---|
| AlS-Se | a | 3 | 3.159 | 3.627 | 2.587 | 2.339 | 2.649 |
| | b | 5 | 3.138 | 3.629 | 2.587 | 2.340 | 2.652 |
| | c | 61 | 3.608 | 3.625 | 2.588 | 2.339 | 2.648 |
| | d | 0 | 3.123 | 3.627 | 2.588 | 2.340 | 2.651 |
| | e | 62 | 3.617 | 3.624 | 2.587 | 2.337 | 2.647 |
| | f | 6 | 3.160 | 3.629 | 2.586 | 2.340 | 2.652 |
| AlS-Te | a | 1 | 3.273 | 3.821 | 2.578 | 2.401 | 2.984 |
| | b | 14 | 3.264 | 3.826 | 2.580 | 2.404 | 2.984 |
| | c | 66 | 3.728 | 3.817 | 2.577 | 2.399 | 2.982 |
| | d | 0 | 3.617 | 3.822 | 2.579 | 2.402 | 2.985 |
| | e | 69 | 3.389 | 3.817 | 2.578 | 2.399 | 2.982 |
| | f | 12 | 3.273 | 3.826 | 2.580 | 2.403 | 2.984 |
| AlSe-Se | a | 4 | 3.156 | 3.741 | 2.579 | 2.462 | 2.674 |
| | b | 2 | 3.070 | 3.744 | 2.580 | 2.462 | 2.678 |
| | c | 72 | 3.652 | 3.736 | 2.580 | 2.460 | 2.672 |
| | d | 0 | 3.089 | 3.744 | 2.580 | 2.462 | 2.678 |
| | e | 73 | 3.663 | 3.737 | 2.580 | 2.460 | 2.671 |
| | f | 4 | 3.124 | 3.744 | 2.579 | 2.462 | 2.678 |
| AlSe-Te | a | 3 | 3.272 | 3.934 | 2.576 | 2.522 | 2.997 |
| | b | 11 | 3.184 | 3.939 | 2.574 | 2.522 | 2.999 |
| | c | 75 | 3.815 | 3.932 | 2.574 | 2.520 | 3.000 |
| | d | 0 | 3.180 | 3.935 | 2.573 | 2.522 | 2.999 |
| | e | 78 | 3.822 | 3.930 | 2.575 | 2.520 | 2.996 |
| | f | 11 | 3.271 | 3.938 | 2.573 | 2.522 | 2.995 |
| AlTe-Se | a | 21 | 3.075 | 3.953 | 2.567 | 2.656 | 2.721 |
| | b | 0 | 2.908 | 3.965 | 2.568 | 2.659 | 2.736 |
| | c | 122 | 3.763 | 3.943 | 2.567 | 2.654 | 2.730 |
| | d | 16 | 3.042 | 3.956 | 2.566 | 2.655 | 2.732 |
| | e | 122 | 3.754 | 3.943 | 2.567 | 2.653 | 2.720 |
| | f | 10 | 3.033 | 3.956 | 2.568 | 2.657 | 2.718 |
| AlTe-Te | a | 7 | 3.307 | 4.126 | 2.566 | 2.705 | 3.027 |
| | b | 7 | 3.220 | 4.136 | 2.566 | 2.707 | 3.033 |
| | c | 100 | 3.951 | 4.121 | 2.565 | 2.702 | 3.030 |
| | d | 0 | 3.212 | 4.129 | 2.566 | 2.704 | 3.031 |
| | e | 101 | 3.965 | 4.119 | 2.566 | 2.701 | 3.023 |
| | f | 12 | 3.285 | 4.131 | 2.565 | 2.706 | 3.023 |

**Table S3.** The energy differences Δ*E* (meV) and interlayer distances *d* (Å) as well as the lattice constants *a* (Å) and bond lengths *L* (Å) of various configurations for $GaX_A/X_B$ vdW heterostructures

| System | Configuration | Δ*E* | *d* | *a* | $L_{M\text{-}M}$ | $L_{M\text{-}X_A}$ | $L_{X_A\text{-}X_B}$ |
|---|---|---|---|---|---|---|---|
| GaS-Se | a | 6 | 3.104 | 3.656 | 2.466 | 2.375 | 2.656 |
| | b | 5 | 3.037 | 3.660 | 2.467 | 2.376 | 2.653 |
| | c | 71 | 3.585 | 3.654 | 2.468 | 2.374 | 2.654 |
| | d | 0 | 3.020 | 3.660 | 2.468 | 2.377 | 2.657 |
| | e | 72 | 3.598 | 3.654 | 2.468 | 2.375 | 2.659 |
| | f | 8 | 3.092 | 3.659 | 2.467 | 2.376 | 2.657 |
| GaS-Te | a | 7 | 3.153 | 3.861 | 2.477 | 2.442 | 2.987 |
| | b | 14 | 3.081 | 3.866 | 2.477 | 2.443 | 2.987 |
| | c | 78 | 3.647 | 3.855 | 2.478 | 2.438 | 2.991 |
| | d | 0 | 3.062 | 3.863 | 2.476 | 2.443 | 2.991 |
| | e | 80 | 3.671 | 3.856 | 2.476 | 2.439 | 2.994 |
| | f | 18 | 3.158 | 3.865 | 2.476 | 2.442 | 2.989 |
| GaSe-Se | a | 5 | 3.068 | 3.765 | 2.464 | 2.487 | 2.683 |
| | b | 0.1 | 2.969 | 3.770 | 2.465 | 2.488 | 2.677 |
| | c | 81 | 3.658 | 3.757 | 2.463 | 2.484 | 2.676 |
| | d | 0 | 3.020 | 3.764 | 2.463 | 2.487 | 2.679 |
| | e | 82 | 3.647 | 3.758 | 2.462 | 2.485 | 2.683 |
| | f | 5 | 3.059 | 3.765 | 2.463 | 2.488 | 2.677 |
| GaSe-Te | a | 8 | 3.169 | 3.959 | 2.474 | 2.548 | 3.001 |
| | b | 11 | 3.081 | 3.967 | 2.472 | 2.549 | 3.003 |
| | c | 92 | 3.747 | 3.954 | 2.470 | 2.544 | 3.003 |
| | d | 0 | 3.083 | 3.963 | 2.472 | 2.548 | 3.004 |
| | e | 93 | 3.732 | 3.954 | 2.471 | 2.544 | 2.999 |
| | f | 18 | 3.160 | 3.965 | 2.473 | 2.548 | 3.003 |
| GaTe-Se | a | 27 | 3.004 | 3.965 | 2.457 | 2.663 | 2.735 |
| | b | 0 | 2.833 | 3.980 | 2.456 | 2.665 | 2.723 |
| | c | 140 | 3.722 | 3.950 | 2.452 | 2.660 | 2.722 |
| | d | 22 | 2.963 | 3.970 | 2.455 | 2.662 | 2.726 |
| | e | 140 | 3.733 | 3.955 | 2.455 | 2.659 | 2.735 |
| | f | 13 | 2.911 | 3.969 | 2.456 | 2.665 | 2.736 |
| GaTe-Te | a | 8 | 3.208 | 4.134 | 2.464 | 2.713 | 3.027 |
| | b | 4 | 3.113 | 4.149 | 2.464 | 2.714 | 3.037 |
| | c | 113 | 3.915 | 4.129 | 2.461 | 2.707 | 3.031 |
| | d | 0 | 3.126 | 4.141 | 2.464 | 2.712 | 3.036 |
| | e | 113 | 3.954 | 4.130 | 2.462 | 2.708 | 3.024 |
| | f | 15 | 3.209 | 4.141 | 2.463 | 2.713 | 3.033 |

**Table S4.** The energy differences Δ$E$ (meV) and interlayer distances $d$ (Å) as well as the lattice constants $a$ (Å) and bond lengths $L$ (Å) of various configurations for $InX_A/X_B$ vdW heterostructures

| System | Configuration | Δ$E$ | $d$ | $a$ | $L_{M\text{-}M}$ | $L_{M\text{-}X_A}$ | $L_{X_A\text{-}X_B}$ |
|---|---|---|---|---|---|---|---|
| InS-Se | a | 8 | 2.963 | 3.823 | 2.799 | 2.543 | 2.695 |
| | b | 0 | 2.862 | 3.829 | 2.798 | 2.544 | 2.692 |
| | c | 89 | 3.562 | 3.814 | 2.798 | 2.541 | 2.691 |
| | d | 1 | 2.876 | 3.825 | 2.799 | 2.544 | 2.694 |
| | e | 90 | 3.562 | 3.816 | 2.800 | 2.541 | 2.697 |
| | f | 6 | 2.947 | 3.824 | 2.798 | 2.543 | 2.696 |
| InS-Te | a | 10 | 3.054 | 4.023 | 2.811 | 2.593 | 3.010 |
| | b | 5 | 2.961 | 4.029 | 2.810 | 2.595 | 3.011 |
| | c | 95 | 3.632 | 4.018 | 2.811 | 2.591 | 3.013 |
| | d | 0 | 2.964 | 4.024 | 2.811 | 2.593 | 3.012 |
| | e | 97 | 3.643 | 4.018 | 2.811 | 2.591 | 3.013 |
| | f | 14 | 3.005 | 4.029 | 2.813 | 2.594 | 3.012 |
| InSe-Se | a | 14 | 2.918 | 3.913 | 2.793 | 2.653 | 2.720 |
| | b | 0 | 2.776 | 3.921 | 2.795 | 2.655 | 2.713 |
| | c | 108 | 3.581 | 3.903 | 2.791 | 2.649 | 2.712 |
| | d | 8 | 2.840 | 3.915 | 2.793 | 2.652 | 2.715 |
| | e | 108 | 3.567 | 3.903 | 2.793 | 2.650 | 2.719 |
| | f | 8 | 2.864 | 3.914 | 2.794 | 2.653 | 2.719 |
| InSe-Te | a | 10 | 3.041 | 4.108 | 2.806 | 2.699 | 3.025 |
| | b | 3 | 2.931 | 4.117 | 2.805 | 2.700 | 3.027 |
| | c | 105 | 3.690 | 4.102 | 2.804 | 2.696 | 3.023 |
| | d | 0 | 2.980 | 4.112 | 2.804 | 2.700 | 3.027 |
| | e | 106 | 3.730 | 4.103 | 2.800 | 2.696 | 3.022 |
| | f | 13 | 3.036 | 4.112 | 2.804 | 2.700 | 3.024 |
| InTe-Se | a | 55 | 2.844 | 4.095 | 2.780 | 2.833 | 2.770 |
| | b | 0 | 2.521 | 4.124 | 2.782 | 2.842 | 2.754 |
| | c | 187 | 3.683 | 4.075 | 2.778 | 2.827 | 2.763 |
| | d | 49 | 2.768 | 4.105 | 2.780 | 2.832 | 2.758 |
| | e | 187 | 3.672 | 4.077 | 2.779 | 2.828 | 2.764 |
| | f | 23 | 2.662 | 4.106 | 2.782 | 2.843 | 2.773 |
| InTe-Te | a | 14 | 3.103 | 4.272 | 2.790 | 2.869 | 3.059 |
| | b | 0 | 2.932 | 4.290 | 2.792 | 2.872 | 3.064 |
| | c | 133 | 3.891 | 4.264 | 2.787 | 2.863 | 3.057 |
| | d | 6 | 3.027 | 4.281 | 2.789 | 2.869 | 3.062 |
| | e | 133 | 3.893 | 4.266 | 2.790 | 2.864 | 3.050 |
| | f | 12 | 3.061 | 4.278 | 2.790 | 2.871 | 3.059 |

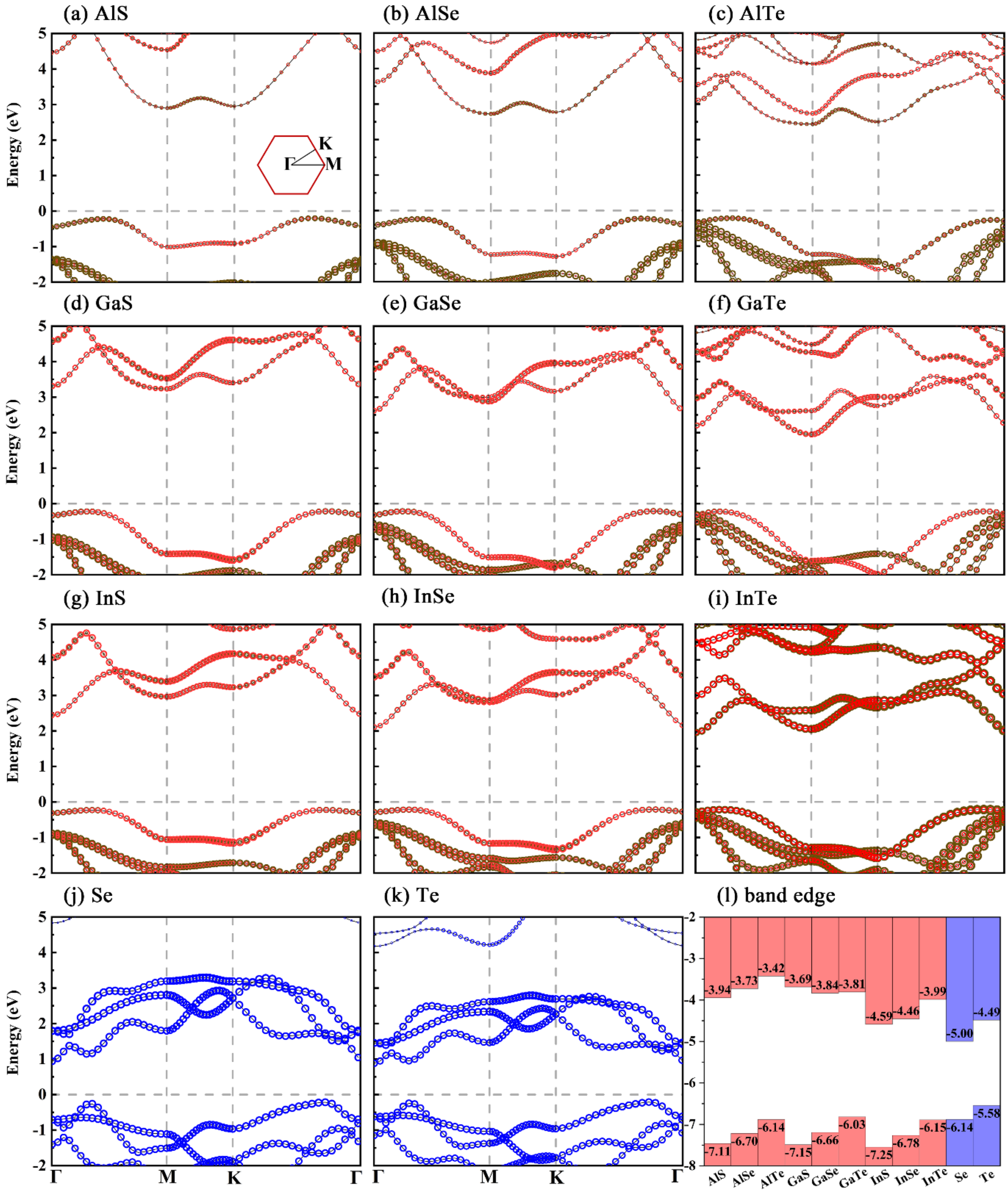


**Figure S1.** Projected band structures of (a) AlS, (b) AlSe, (c) AlTe, (d) GaS, (e) GaSe, (f) GaTe, (g) InS, (h) InSe, (i) InTe, (j) Se and (k) Te monolayers by HSE06 hybrid functional method. The red, brown, blue circles represent the projected specific gravity of M, $X_A$, $X_B$ atoms, respectively. The first Brillouin zone with high-symmetry points are shown in the inset of (a). (l) The band edge alignments of these monolayers.
